# Artifact detection in fluorescence microscopy using convolutional autoencoder

**DOI:** 10.1038/s41598-025-18943-6

**Published:** 2025-09-12

**Authors:** Fabian Rehn, Marlene Pils, Tuyen Bujnicki, Oliver Bannach, Dieter Willbold

**Affiliations:** 1 Institut für Physikalische Biologie, Heinrich-Heine-Universität Düsseldorf, Universitätsstr. 1, 40225 Düsseldorf, Germany; 2 Institute of Biological Information Processing (Structural Biochemistry: IBI-7), Forschungszentrum Jülich GmbH, Wilhelm-Johnen-Straße, 52428 Jülich, Germany; 3 attyloid GmbH, Merowingerplatz 1A, 40225 Düsseldorf, Germany

**Keywords:** Microscopy, Fluorescence imaging, Data publication and archiving, Image processing, Machine learning

## Abstract

To ensure analytical accuracy in fluorescence microscopy image analysis, robust artifact detection is essential. For large datasets or time-sensitive analyses, automation is advisable, as it not only reduces time and costs but also eliminates human bias and enhances reproducibility. Although artificial intelligence is commonly employed for artifact detection, it is typically limited to recognizing artifact types that have been previously learned, often necessitating large training datasets. This study proposes an approach for an automated detection of previously unseen artifacts without the need for a training set of artifact-laden images. Multiple datasets were assembled using images generated by our surface-based intensity distribution analysis (sFIDA) technology during different experiments. A convolutional autoencoder was trained on a dataset of artifact-free images to reproduce preprocessed images accurately. Artifact-laden images are subsequently detected by computing the difference between the input and output of the model, with increased discrepancies indicating the presence of artifacts. The proposed model is capable of classifying artifacts across different datasets with an average accuracy of 95.5%. Additionally, the model was able to detect unseen artifacts of various types, including differences in cause, structure, size and intensity. The findings demonstrate that convolutional autoencoders provide a lightweight, but effective method for detecting artifact-laden images. While the method was tested only on sFIDA images, its design, which does not rely on an artifact specific training set, suggests potential for use across various microscopy techniques.

**Supplementary Information:**

The online version contains supplementary material available at 10.1038/s41598-025-18943-6.

## Introduction

In all types of microscopic imaging, the detection of artifacts is essential for ensuring analytical accuracy. This is particularly critical in quantitative fluorescence microscopy, where artifacts can markedly distort assay readouts. There are various types of artifacts, which can be categorized into three main groups: microscope-related artifacts, material-related artifacts, and sample-related artifacts. The first type is highly dependent on the type of microscopy used. For example, striping artifacts are common in light-sheet fluorescence microscopy^1^, while fixation artifacts affect stochastic optical reconstruction microscopy^2^. Overlapping structures and fluorescent impurities introduce artifacts in single-molecule localization microscopy^3^ and path-length differences between sample and standard can cause artifacts in fluorescence lifetime imaging microscopy^4^. Air bubbles in the immersion oil can cause artifacts in TIRF microscopy^5^ and intravital fluorescence microscopy is frequently confronted with motion artifacts^6^. Additionally, artifacts caused by motion, focus issues and contamination represent broader challenges that can arise across various imaging methods. Material-related artifacts arise from hardware issues, such as scratches on the plate or are related to glue, which can induce autofluorescence or lead to motion artifacts if the glue fails. Sample-related artifacts can be caused by clustering of fluorescent probes^7^, contamination with dust leading to autofluorescence or nonspecific binding^8^, bacterial contamination, intrinsic fluorescence of the sample^9^, oversaturation or due to incorrect preparation or collection of samples^10^. Some microscope-related artifacts, such as optical aberrations, might be detected directly by the microscope software. However, this is not the case for material or sample-related artifacts. Manual inspection of images to identify and discard those containing artifacts is often an inefficient solution, especially for assays that generate large volumes of data or where quick data evaluation is desirable. Besides the excessive time required, a manual approach is susceptible to human error, and even worse, human bias. Since artifacts are not always easy to identify, leaving room for interpretation, manual evaluation also reduces the reproducibility of the analysis. Ultimately, these factors impede the efficient up-scaling of assay throughput.

One example of a high throughput assay based on fluorescence microcopy is the surface-based fluorescence intensity distribution analysis (sFIDA) technology. sFIDA was developed to quantify various types of protein oligomers which serve as biomarkers for different protein misfolding diseases^11–14^. This method typically generates at least 100 images per sample, resulting in large amount of data, making manual artifact detection the bottleneck of the analysis. As an assay quantifying fluorescence microscopy signals, artifacts can markedly distort assay readouts. As the removal of artifacts would reduce the remaining image area, potentially affecting the results of the quantitative analysis, only the detection of artifacts for the purpose of excluding affected images from the analysis is necessary. Consequently, it is sufficient to determine the presence of artifacts at the image level, while identifying the specific pixels associated with the artifact is not relevant. For reasons of quality, time, and cost, an automated solution for the detection of images containing artifacts is preferable.

Automatic approaches for artifact detection typically rely on kernel-based methods^15^, or leverage machine learning algorithms. In the latter, convolutional neural networks, which focus on the detection of predefined and learned artifact structures^16,17^, are commonly used, sometimes in conjunction with segmentation^18^. While specializing in predefined artifacts is an effective approach, given that machine learning techniques can be used to learn and recognize structures in images with high accuracy, it does not address all potential issues. By limiting detection to specific learned artifact structures, previously unknown artifact types, such as those arising from contamination or compromised material, may remain undetected. This is particularly problematic in the context of scaled, automated workflows, as it could lead to persistent distorted results until the issue is noticed. As the number of possible artifact appearances is almost unlimited due to the wide variety of sources, and considering that a large amount of training data is recommendable to achieve the highest possible accuracy^19^, constructing such a diverse dataset would be highly impractical.

Training a model without a large dataset of artifact-laden images is possible by designing the model to recognize authentic signals rather than artifacts. Every structure that cannot be confirmed to be an authentic signal, consequently, can be assumed to be an artifact and can be ignored during subsequent analysis. The circumstance that real signals are often easier to identify, based on their known characteristics, and that a large volume of training images is easy to collect further supports this approach. One possible implementation is the combination of segmentation techniques and deep learning classification algorithms. Single structures are first identified and then classified individually. Depending on the number of elements to be identified and classified, the method is time-consuming, and models involved are usually complex. Furthermore, pixel-level labels are usually required, which substantially increases the effort required to create a dataset. Although there are segmentation approaches that allow image-level labelling^18^, they usually result in a decline in performance. Another approach is the utilization of a convolutional autoencoder (CAE). CAEs are closely related to convolutional neural networks, with the key distinction that they are typically symmetrically designed to first reduce the dimensionality of the input and subsequently reproduce it. This reduction process focuses on learning essential patterns^20^, making it particularly useful for denoising images^21^.

CAEs are already employed for denoising medical and biological image data^22,23^ and for the removal of certain types of artifacts, such as stripes^24^. However, artifact removal is feasible only for artifacts covering small areas, where minimal reconstruction is required. In cases where artifact removal and subsequent reconstruction are unsuitable for image analysis, artifact-laden images should be identified and excluded. To this end, CAE principles can be adapted for artifact detection. This involves training a CAE solely on artifact-free input with the objective of precisely reproducing the input after dimensionality reduction. Consequently, the CAE should exhibit low error in reproducing artifact-free input while showing higher deviation for input containing artifacts. As a result, the discrepancy between the CAE’s input and output images is expected to be greater for images with artifacts. Based on this reproduction error artifacts can be detected. An equivalent approach has already been applied for anomaly detection in MRI images^25^. The requirements for the CAE thus depend strongly on the specific application and may necessitate varying levels of complexity based on the intricacy of the structures involved.

The objective of this work is to demonstrate based on sFIDA-generated images that CAEs can be effectively used for the detection of artifact-laden images without requiring artifact-laden images for training.

## Methods

### sFIDA

**Fig. 1:**
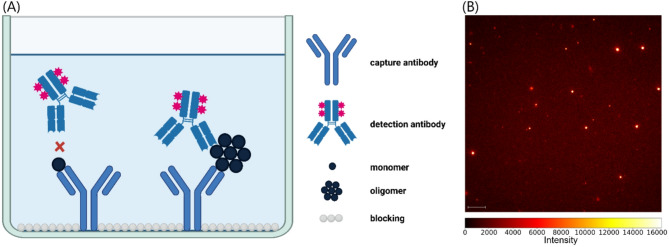
(**A**) Schematic representation of sFIDA technology. The capture antibody is immobilized on the glass surface. Both oligomers and monomers can bind to the capture antibodies. However, only oligomers can be detected by fluorescence signalling, as the epitope of the monomers is masked by the capture antibody, preventing fluorescently labeled detection antibodies from binding. Created with BioRender.com. (**B**) Example of sFIDA-generated image, showing signals from Aβ-coated SiNaPs. The image was colorized and contrast has been increased to enhance visibility. The scalebar is equal to 10 μm.

Previously, we developed the sFIDA platform technology, which enables monomer-insensitive quantification of protein oligomers immobilized on the glass surface of a microtiter plate^26^. Individual oligomers are detected and quantified by using fluorescent antibody probes and imaging with a Leica AM total internal reflection fluorescence (TIRF) microscope, which produces 1000 × 1000 pixel, 14-bit grayscale images. In these images, each pixel has a grayscale value that reflects the intensity of the fluorescent signal. In the following, it is referred to as intensity. Pixels, or clusters of neighboring pixels, with intensities distinguishable from the background are referred to as signals. Figure 1 illustrates the basic principle of sFIDA, along with an example of an artifact-free sFIDA image. Furthermore, we have developed silicon nanoparticles (SiNaPs) that serve as artificial targets for the sFIDA assay and can be used to calibrate the assay readout^27^. All images presented in this work were generated using sFIDA assays.

Protein oligomers, typically 2 to 10 nm in size, are smaller than the approximately 120 nm resolution of the TIRF microscope. As a result, oligomers occupy less space than a single pixel in a TIRF microscopy image, preventing sFIDA from capturing structural details of individual oligomers. Larger oligomers, though below the resolution limit, can emit enough fluorescence to affect neighboring pixels. With more available binding sites, these larger oligomers bind more labeled antibodies, thereby increasing fluorescence intensity and creating a broader emission radius in sFIDA images. When signals from nearby oligomers overlap, complex signal shapes may emerge.

Artifacts in sFIDA can have various origins. These include well-known sources of TIRF microscopy, such as air bubbles and oil rings, which, however, can be mostly avoided and do not significantly affect the sFIDA assay. Since they do not generate signals themselves and the signals from the detection antibodies used are often not attenuated sufficiently, their impact on analysis remains minimal. In the context of quantitative image analysis, artifacts that generate fluorescence signals are of greater concern. This is particularly relevant in cases of contamination, as they can enable nonspecific binding of detection antibodies or exhibit autofluorescent properties. As a result, artifacts may produce signals whose area can exceed that of authentic signals generated by detection antibodies bound to the analyte, leading to a distortion in quantitative evaluation. Additionally, artifacts can arise in the form of numerous authentic signals, clustered as agglomerates in limited space. Given the virtually unlimited variations in the appearance of material defects, contamination-related nonspecific bindings, and matrix components, a highly diverse range of artifacts can occur, depending on the sample matrix, antigen, and antibody used. The central challenge in artifact detection within the sFIDA assay is therefore the development of a method that operates reliably regardless of these factors.

### Data preparation

To avoid using limited model capacity on learning random background noise patterns, background was removed from the images. The challenge in distinguishing signal from background lies in the variance of the background noise. A Gaussian blur with a kernel size of 5 × 5 pixels was applied, to align the intensity of individual pixels to their surroundings, thereby reducing variance while preserving strong signals or those that span multiple pixels. Since most of the area in a typical sFIDA-generated image is background, resulting in low intensities, signal can be effectively separated from the background using an intensity threshold. For this, a value equal to the mean intensity of an image plus five standard deviations has proven effective. Intensities below this threshold are set to zero, while those above remain unchanged.

The processing time in a convolutional network is influenced by several factors, one of which is the size of the input. The number of operations required in each layer is proportional to the size of the input. Consequently, reducing the image size from 1000 × 1000 pixels to 500 × 500 pixels leads to a considerable decrease in processing time. To realize this, a 2 × 2 block reduction technique was applied, where each block was aggregated into a single pixel by calculating the blocks mean intensity. Due to the prior application of a threshold and the resulting substantial difference between signal and background intensities, signals are preserved despite the size reduction. Finally, the images are normalized to a signal strength between zero and one to facilitate the fitting process and subsequent predictions by the convolutional autoencoder.

The Gaussian blur is implemented using the Python library *OpenCV (version 4.6.0)*. Size reduction was performed using python library *scikit-image (version 0.23.1).*

### Convolutional autoencoder

In sFIDA-generated images, the signals lack complex structural characteristics but display large variability in signal strength (Fig. 1). This makes a convolutional autoencoder (CAE) with a relatively small number of layers and a moderate number of filters a suitable choice. To minimize processing time and mitigate the risk of overfitting, hyperparameter optimization, encompassing both the network architecture, as well as the activation and loss functions used, was initiated with a single layer and a minimal number of filters, which were progressively increased until no further performance improvements were observed.

**Fig. 2:**
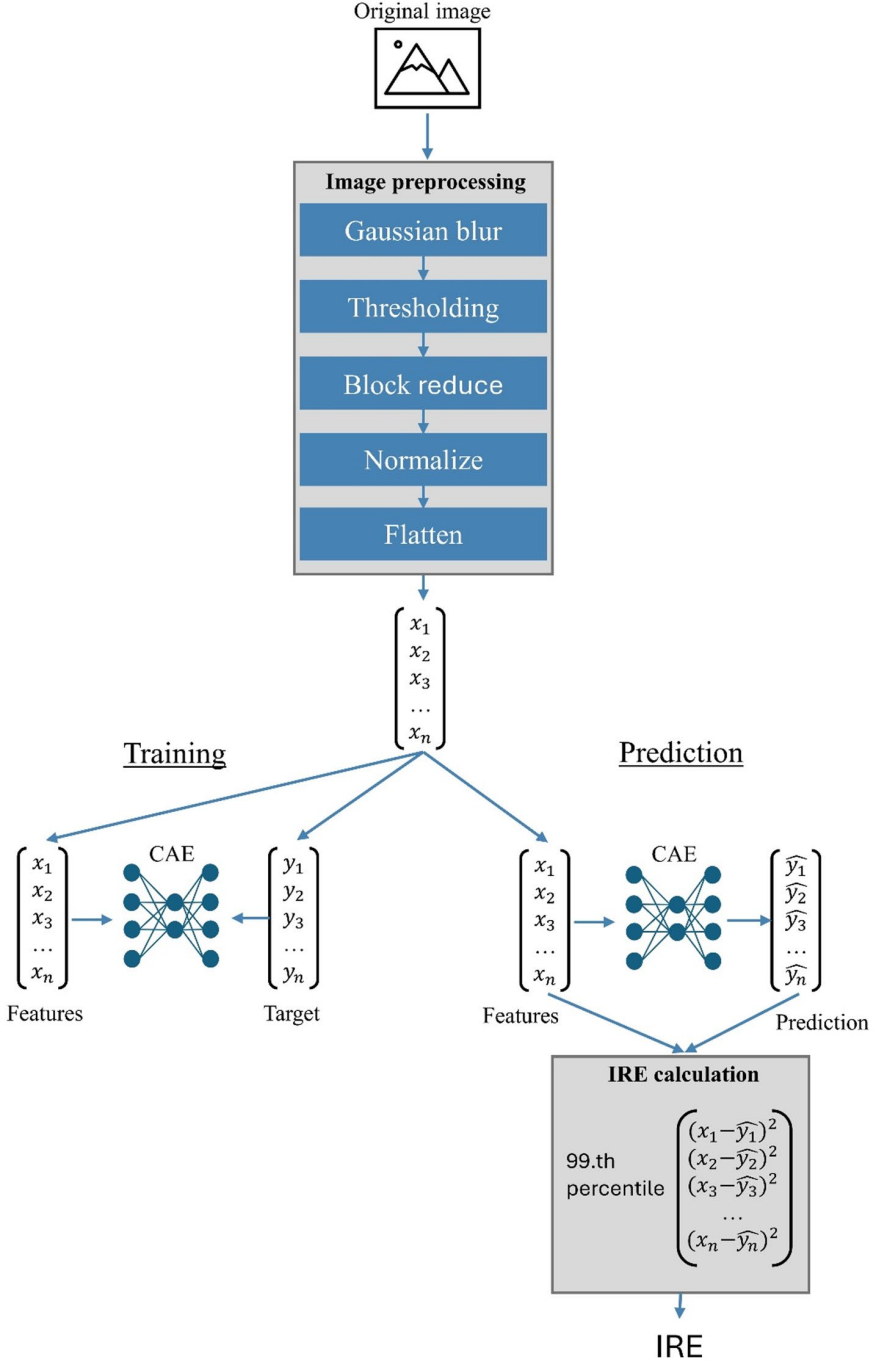
Schematic representation of the processing of a single image. It should be noted that an image is either used for model training or its IRE is calculated.

The CAE was trained using 224 artifact-free sFIDA images of artificial Aβ-coated SiNaPs from dataset 1, which were previously manually labeled (see below). These artificial samples offer higher reliability in artifact identification compared to human specimens, while maintaining identical signal characteristics, thereby ensuring the highest data quality during training. To train the model to reproduce the input as accurately as possible, the input itself was also used as the target during training (Fig. 2). After each training epoch, the model’s performance was evaluated, to determine whether a satisfactory training state had been achieved or if further training would be beneficial. For this purpose, the validation set consisting of the remaining 96 artifact-free and 87 artifact-laden manually labeled images from dataset 1, was used. For each image, the mean squared error (MSE) between the model input and output was calculated. Afterwards, the MSE of artifact-free images as well as the percentual difference between artifact-free and artifact-laden images were used as a metric. Due to the small size of the model and training dataset, training for only eight epochs was sufficient (Supplementary Fig. 1). Hyperparameter optimization was also carried out based on these metrics. Table 1 presents the architecture of the CAE. This approach was chosen because a minimal MSE between artifact-free images and Model output did not necessarily lead to the best differentiation between artifact-free and artifact-laden images.

The CAE was implemented using Python’s Keras package, version (2.3.1).

**Table 1:** The individual Keras layers of the CAE and their specifications are listed below.

Layer	Filter	Kernel Shape, strides	Activation function	Output Shape
Input Layer				(500,500,1)
Conv2D	126	(2,2)	RELU	(500,500,126)
MaxPooling2D		(2,2)		(250,250,126)
Conv2D	256	(5,5)	RELU	(250,250,256)
MaxPooling2D		(2,2)		(125,125,256)
Conv2DTranspose	256	(5,5),2	RELU	(250,250,256)
Conv2DTranspose	126	(2,2),2	RELU	(500,500,126)
Conv2D	1	(2,2)	RELU	(500,500,1)

### Image reproduction error

To assess whether an image contains artifacts, the input and output images of the CAE are compared. Since the model has been trained exclusively on artifact-free images, it is not expected to accurately reproduce artifacts. The squared error is calculated for each pixel between the input image $\:I$ and the output image $\:O$ of the CAE. Given that artifacts typically occupy only a small portion of the image, most pixels should exhibit squared errors in an inconspicuous range. As a result, smaller artifacts may not be detected if using a mean deviation across all pixels as metric. To enhance sensitivity to small artifacts, the image reproduction error (IRE) is defined as the 99.99th percentile of the squared pixel errors (Eq. 1).

1 $$\:IRE=\:Percentile\left({\{\left({I}_{x,y}-{O}_{x,y}\right)}^{2}\:\right|x=1,\dots\:,500\:,\:y=1,\dots\:,500\:\},0.9999)$$

To assess whether an image contains artifacts or not, a threshold must be established beyond which an image is considered as artifact-laden. A careful balance is required to minimize the false-negative classification of artifact-laden images as artifact-free while also ensuring that as few artifact-free images as possible are classified as false-positives and consequently discarded. In this work, we adjusted the 1.5 interquartile range method for outlier detection to achieve this balance. The threshold is defined as the 3-fold distance between the 0.25 and 0.5 quantiles of all IRE, added to the 0.5 quantile of the IRE for each dataset. By using the distance between the 0.25 and 0.5 quantiles, it is ensured that the threshold is not influenced by artifacts, even in datasets with a high artifact load. Since the distance used is half of the usual interquartile range, the multiplier is doubled as compensation. It is assumed that potential artifacts in images below this threshold are negligible and do not significantly affect the analysis results.

Figure 2 shows the entire processing pipeline of the model.

### Datasets

Six different datasets were used for the training, validation, and testing of the developed model. All datasets originate from previously evaluated experiments which were not conducted specifically for this work. Each non-artificial dataset includes sFIDA images from blinded selected individuals, i.e. independently of further information, such as demographic information or clinical diagnoses. During the selection process, care was only taken to ensure that a sufficiently large number of artifact-laden image was included. Additionally, images that could not be definitively classified as either artifact-free or artifact-laden using the four-eyes principle were excluded, resulting in varying numbers of images across the datasets. The specifications of the datasets are listed below.

- ***Dataset 1***: sFIDA images of artificial Aβ-SiNaPs in two-fold dilution in buffer ranging from 128 to 2 fM. The assay used is described by Blömeke et al. (2024b)^14^. The set comprises 320 artifact-free images and 87 artifact-laden images. This dataset is employed for training and validating the models.
- ***Dataset 2***: sFIDA images of human Aβ oligomers in the plasma of 5 individuals measured by Blömeke et al. (2024b)^14^. This dataset was generated in the same experiment as the artificial SiNaPs presented in **Dataset 1**. The set comprises 318 artifact-free images and 97 artifact-laden images. It will be used to investigate whether a model trained on artificial signals can be transferred to human data, enabling unbiased artifact detection in human samples.
- ***Dataset 3 + 4***: Additional images generated by Blömeke et al. (2024b) using the same assay as in the **Dataset 1** and **2** but in different experiments including plasma of 5 different individuals each^14^. The sets contain 276 and 308 artifact-free images, respectively, and 97 and 61 artifact-laden images, respectively. These sets will be used to examine whether a model trained on a different experiment of the same assay can be applied to other experiments, allowing the use of a single model for scaled applications.
- ***Dataset 5***: sFIDA images of human IAPP oligomers in plasma of 10 individuals generated by Rehn et al. (2024)^13^. The number of individuals was doubled to compensate for the lower number of artifacts in the dataset. This set includes 943 artifact-free images and 56 artifact-laden images. It will be used to determine whether the model can be applied to assays using the same matrix but targeting different antigens, allowing a single model to be used across a broad range of assays.
- ***Dataset 6***: sFIDA images of human Aβ in CSF of 10 individuals generated by Blömeke et al. (2024a)^11^. The number of individuals was doubled to compensate for the low number of artifacts in the dataset. This set includes 721 artifact-free images and 25 artifact-laden images. It will be used to investigate whether the model can be transferred to other matrices and target different antigens, enabling a universal application of a single model.

All methods were performed in accordance with the relevant guidelines and regulations (Declaration of Helsinki).

### Reference Model

To assess the performance of the proposed CAE in relation to an established model, a CNN was used as a reference. The VGG-16 architecture^28^, which consists of 16 layers exhibits a higher level of complexity compared to the used CAE. This architecture was chosen due to its demonstrated effectiveness in various applications involving the analysis of medical microscopy imaging data^29,30^. Moreover, it is recognized for its broad applicability and performance.

The CNN was implemented to classify images directly as either artifact-free or artifact-affected. For this purpose, the labeled images of Dataset 1 were utilized for training. Unlike the CAE, both artifact-free and artifact-affected images were directly incorporated into the training process. The preprocessed images were used in order to achieve the highest possible level of comparability. To be able to use these as input, they were scaled to the input shape of the VGG-16. The model was trained for 25 epochs (see supplementary Fig. 2), with the number of epochs determined based on the best balance between accuracy and loss achieved on the validation dataset. During training, an identical number of artifact-free and artifact-affected images was used in each epoch. Around 25% of the artifact-affected images were used as validation set.

This direct classification approach not only facilitates a comparative performance analysis but also enables an evaluation of the key hypothesis that algorithms trained in this manner fail when confronted with previously unseen artifacts.

### Statistics

A two-tailed t-test was performed to evaluate the basic assumption of this study that artifact-laden images lead to higher IRE values than artifact-free images. The t-test was chosen because, given the size of the datasets, the assumption of normality of IRE values based on the central limit theorem was appropriate.

To further investigate the potential of IRE for distinguishing between artifact-free and artifact-laden images, an exploratory ROC analysis was performed, to calculate ROC curves and their corresponding area under the curve (AUC). For this a bootstrapping approach was used, randomly generating 10,000 subsets per dataset. To avoid a bias by imbalance between the classes (artifact-free and artifact-laden), subsets were balanced. Based on the IRE values of artifact-free and artifact-laden images a logistic regression was applied on each subset to generate a ROC curve and calculate the corresponding AUC. The mean value of the 10,000 individual AUC values was then calculated for each data set.

Logistic regression was used to generate the ROC curves, and both the logistic regression and ROC curve calculations were performed using the Python package scikit-learn (version 1.0.2). The t-tests were performed using Python package scipy (version 1.7.3).

## Results

**Fig. 3:**
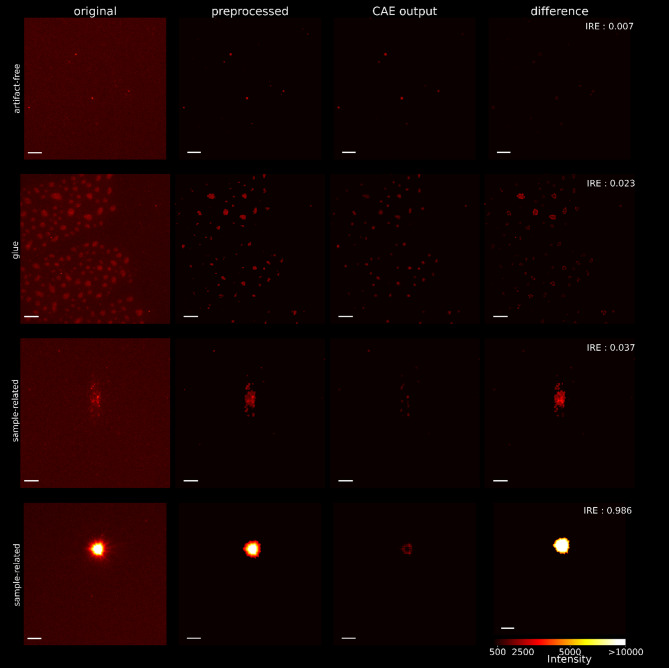
Examples illustrate the image processing by the preprocessing steps and the CAE. The first column shows unprocessed zoomed-in sections from some of the images in Fig. 4. The second column displays these sections after applying the preprocessing steps. The third column shows the output of the CAE. The fourth column presents the absolute difference between the input and the output of the CAE. The first row shows the processing of an artifact-free image, while the remaining rows present artifact-laden images. The scalebar is equal to 5 μm.

The aim of this study was to investigate whether CAEs can be used to identify sFIDA images containing artifacts. To this end, sFIDA images were first preprocessed, and a CAE was subsequently trained to reproduce artifact-free images. The trained CAE was then applied to both artifact-free and artifact-laden images. Figure 3 presents example images from the different stages of the data pipeline. It shows that the CAE reproduces authentic signal with minimal error, while its performance declines for images containing artifacts, resulting in an increased IRE. This increase in IRE is more pronounced for artifacts that significantly differ from the authentic signal compared to those with only minor deviations.

**Fig. 4:**
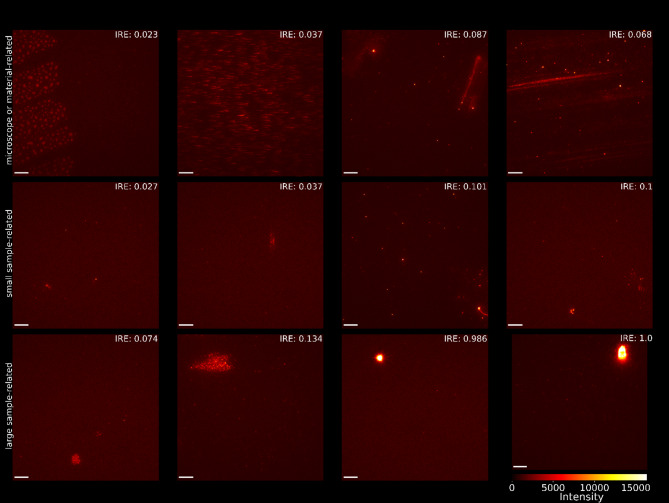
Examples of sFIDA generated images. The first row contains images with microscope or material-related artifacts, such as glue on the plate, motion artifacts and scratches on the plate. The second row contains small-sized sample-related artifacts. The third row contains larger sample-related artifacts. The scalebar is equal to 10 μm.

Figure 4 shows a larger set of original images containing artifacts, along with the IRE values resulting from applying the CAE. The selected images represent all three types of artifacts: microscope-related artifacts, material-related artifacts, and sample-related artifacts. It is noticeable that larger or stronger signals lead to a higher IRE compared to more subtle artifacts.

### Image reproduction errors are significantly increased in artifact-laden images

**Fig. 5:**
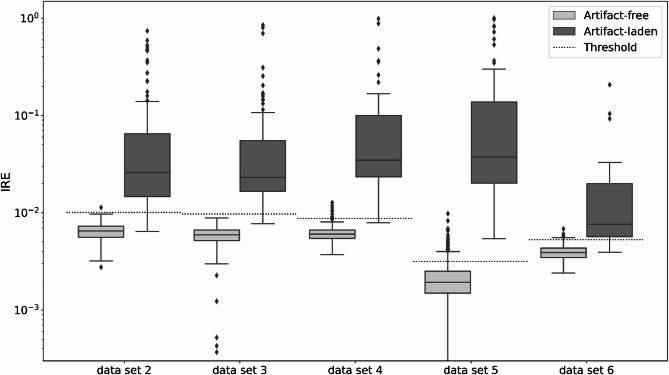
For each dataset, the IRE of the images with and without artifacts are shown. Note: Logistic scaling was used. The dotted line represents the dataset-specific threshold, which will be used to classify images as artifact-free or artifact-laden. The considered range was limited downwards due to irrelevance.

To assess the suitability of IRE values derived from the CAE for detecting artifact-laden images, images from all dataset were manually classified as either artifact-free or artifact-laden and subsequently processed using the methods previously described. IRE values for artifact-laden images were consistently higher across all datasets compared to artifact-free images (Fig. 5). Furthermore, the IRE values for artifact-free images from the datasets 2,3 and 4, which were based on different experiments using the same assay, were at comparable levels. In contrast, the IRE values for artifact-free images from other datasets, which were based on different assays, were at varying levels.

Table 2 presents the *P*-values of a two-sided t-test between IRE of artifact-free and artifact-laden images for each dataset. With a maximum *P*-value of 6.78e-13, the values are far below the significance level of 0.05. Consequently, the differences are highly significant across all datasets. To further evaluate prediction accuracy, ROC curves and their corresponding AUCs were generated repeatedly for randomized subsets of each dataset. The average AUC across the individual datasets ranged from 0.9644 to 0.9998.

**Table 2:** For each dataset, the differentiability between the IRE values of images with and without artifacts is shown using the P-value of a two-sided t-test, along with the AUC score of an ROC curve.

Dataset	*P* Value t-Test	AUC-Score
2	1.67e-20	0.9841
3	6.78e-13	0.9989
4	7.90e-19	0.9986
5	1.49e-68	0.9998
6	8.41e-34	0.9644

### IRE-based classification

After demonstrating that the IRE has potential for accurate detection of artifact-laden images, the threshold described above was applied to classify the samples as artifact-free or artifact-laden. For all test sets, an average accuracy of 95.5% was achieved, indicating that the chosen threshold is effective in distinguishing between artifact-laden and artifact-free images (Table 3). Artifacts are detected with a sensitivity of 94.2% on average. The mean specificity value of 96.1% indicates that artifact-free images are not excessively excluded. Dataset 6, based on CSF instead of plasma samples, yields the lowest values, especially in terms of sensitivity. To enable further evaluation, supplemental Table 1 presents a confusion matrix.

When comparing the performance of the CAE with that of the reference model (CNN), clear differences become evident. While the CNN correctly classifies all artifact-free images, achieving a specificity of 100%, it identifies only 46.8% of the artifacts on average, performing worse than the CAE. However, since the datasets contain more artifact-free than artifact-affected images, the overall accuracy of the CNN is only slightly lower than that of the CAE.

**Table 3:** For each dataset, the sensitivity, specificity and accuracy of classification of both models are shown.

Dataset	Sensitivity		Specificity		Accuracy	
	CNN	CAE	CNN	CAE	CNN	CAE
2	49.5%	88.7%	100%	99.7%	88.2%	97.1%
3	40.2%	95.9%	100%	100.0%	84.5%	98.9%
4	62.3%	98.4%	100%	96.8%	93.8%	97.0%
5	66.1%	100.0%	100%	85.9%	98.1%	86.7%
6	16.0%	88.0%	100%	98.2%	97.2%	97.9%
Mean	46.82%	94.2%	100%	96.1%	92.36%	95.5%

## Discussion

In this study, we have demonstrated that CAEs are a powerful tool for identification of artifacts in imaging-based methods, such as the sFIDA technology. The image reconstruction error between input and output of CAEs can effectively discriminate between artifact-free and artifact-laden images, making it a robust feature for classification. We showed that a classification, based on dataset-specific thresholds, achieves high mean accuracy of 95.5% in categorizing these images. Furthermore, the probability of detecting artifacts increases with increasing structural deviation from authentic signals (Supplementary Fig. 3). This makes strong distortions of the readouts and consequential the statistics, due to single artifacts less likely. The method’s effectiveness across various artifact types in diverse assays with different targets and matrices, without requiring model retraining, highlights its broad applicability.

A further major advantage of the method is, that the detection is not limited to previous learned artifacts, consequently leading to a small size of the training set, requiring only manually labeled, easily captured, artifact-free images for training and only a few artifact-laden images for validation. This versatility suggests that our approach is not only valuable for scaling existing assays but also facilitating the development of new ones. In contrast, other approaches necessitate artifact images for training and are constrained by the specific artifact types present in the training dataset or by the existence of specific expert models^8,9,21,22^. Since artifact images are not required for the training dataset, the data augmentation step, commonly involving transformations such as image rotation to facilitate the detection of identical artifacts across various orientations, becomes unnecessary, thereby accelerating the training process.

Other approaches, which were considered as very accurate, including network-based methods and support vector machines, achieved accuracies ranging from 91.25% to over 99%^8,9,21,22^. It is important to note that these values are not necessarily directly comparable. This is due to variations within the datasets, including factors such as the extent of noise, the complexity of non-artifact structures, the size and detection complexity of artifacts, as well as operational differences, such as pixel-level versus image-level analysis. However, the fact that the achieved accuracy of the presented method falls within the range considered as highly accurate in other studies on artifact detection in microscopy images reinforces our assessment of its suitability for the intended application. This assessment is further supported by the fact that the datasets used in this work contained comparatively small artifacts of different types, making their detection more challenging, and that the CAE achieves, on average, higher accuracy than the reference model. It must be also considered that, in the context of artifact detection, a small proportion of false positives or false negatives is generally regarded as inevitable, particularly for artifacts that are not readily detectable due to their size^21^. Finally, the low sensitivity of the reference model supports the previously stated assumption that an approach based on learning artifacts is only practical with a comprehensive training dataset and when artifacts exhibit only limited variations to those in the training set.

A limitation of the threshold-based method for classification into artifact-free or artifact-laden categories is the possible influence of artifacts on the threshold itself. This issue arises if more than 50% of the images contain artifacts. For sFIDA, this is not a problem as the usual proportion of artifact-laden images is much lower. For other applications, a high occurrence rate of artifact images would result in an elevated mean IRE level. Since different experiments of the same assay have yielded comparable IRE values, the problem could be detected through the discrepancy of mean IRE levels. Furthermore, given the comparable IRE levels of artifact-free images for different experiments of an assay, it would be feasible to establish an assay-optimized and specific threshold to effectively mitigate this unlikely, yet potential, issue. Another limitation of the proposed method is its inability to detect artifacts with very low signal intensity, as these may be removed during the background removal step in preprocessing. As a result, such artifacts cannot be detected by the CAE (Supplementary Fig. 4). In addition, depending on the threshold value for the IRE, the model may not be able to detect agglomeration artefacts if these consist of individually authentic signals that are distant to each other. However, especially the artifacts with very low intensity are not particularly relevant in case of sFIDA, as background noise is also removed during the quantitative analysis of signals. Even if such removal does not occur for analysis, it can be assumed that artifact-laden images, whose IRE does not significantly differ from that of artifact-free images, would have only a negligible impact on the overall analysis, as the IRE increases with the strength and size of the artifact.

To further enhance the accuracy of the method in the future, exploring additional techniques for generating an optimal threshold will be beneficial. Additionally, investigating the potential of incorporating supplementary features and utilizing meta-learners for classification may also be of interest. Since no signal structures are visible for sFIDA images, the presented method is likely transferable to different kinds of assays, operating sub-resolution. Examples of this may include analyses of individual cells or molecules^31,32^. Whether the method can also be applied to microscopy images with more complex structures cannot be determined with certainty based on the available data. However, since autoencoders have already been successfully used for noise removal in such contexts, demonstrating that these more complex structures can be learned and reproduced^23,33,34^, the assumption seems reasonable. Nevertheless, the architecture of the CAE would need to be adapted to the specific complexity of the images. Another important aspect for future investigation is the model’s tolerance to signal variability. In the case of sFIDA, the model trained on artificially generated signals also performed well for different human analytes, which, exhibited limited variations in their generated signals. However, it remains uncertain how the model would react to high variation of signals in applications involving more complex signals. While adjusting the model architecture can help modulate its tolerance to variations, this would likely also increase its susceptibility to artifacts. The extent of this effect, however, is highly application-dependent and should be assessed within the specific context.

## Conclusion

We have demonstrated that CAEs represent a promising approach for fully automated and effective artifact detection. Our results show that the method, exemplified by sFIDA images, delivers high performance in distinguishing between artifact-laden and artifact-free images, irrespective of the antibody used or the matrix employed. However, in the future, this method should be tested on other types of microscopy images to verify its applicability across different microscopy modalities.

## Supplementary Information

Below is the link to the electronic supplementary material.

Supplementary Material 1

## Data Availability

The authors confirm that the data supporting the findings of this study are available within the article and its supplementary materials. The original images as well as the preprocessed images are freely accessible online https://doi.org/10.5281/zenodo.13884860.
