# Supplementary material for: Artifact detection in fluorescence microscopy using convolutional autoencoder
Source: Sci Rep. 2025 Sep 12;15:32482. doi: 10.1038/s41598-025-18943-6 (PMC12432141; doi:10.1038/s41598-025-18943-6)
Supplement: Supplementary file 1 — Supplementary Material 1 [file 41598_2025_18943_MOESM1_ESM.docx]

# Supplement

Artifact detection in fluorescence microscopy using convolutional autoencoder

Fabian Rehn^1,2,3^, Marlene Pils^3^, Tuyen Bujnicki^2^, Oliver Bannach^2,1,3^, Dieter Willbold^2,1,3^

^1^Institut für Physikalische Biologie, Heinrich-Heine-Universität Düsseldorf, Universitätsstr. 1, 40225 Düsseldorf, Germany.

^2^Institute of Biological Information Processing (Structural Biochemistry: IBI-7), Forschungszentrum Jülich GmbH, Wilhelm-Johnen-Straße, 52428 Jülich, Germany.

^3^attyloid GmbH, Merowingerplatz 1A, 40225 Düsseldorf, Germany.

|  | Predicted Artefact-laden | Predicted Artefact-free |
| --- | --- | --- |
| Dataset 2- Actual Artefact-laden | 88.7% | 11.3% |
| Dataset 2 - Actual Artefact-free | 0.3% | 99.7% |
| Dataset 3 - Actual Artefact-laden | 95.9% | 4.1% |
| Dataset 3 - Actual Artefact-free | 0.0% | 100.0% |
| Dataset 4 - Actual Artefact-laden | 98.4% | 1.6% |
| Dataset 4 - Actual Artefact-free | 3.2% | 96.8% |
| Dataset 5 - Actual Artefact-laden | 100.0% | 0.0% |
| Dataset 5 - Actual Artefact-free | 14.1% | 85.9% |
| Dataset 6 - Actual Artefact-laden | 88.0% | 12.0% |
| Dataset 6 - Actual Artefact-free | 1.8% | 98.2% |

**Sup. Table 1**: Confusion Matrix of classification results.


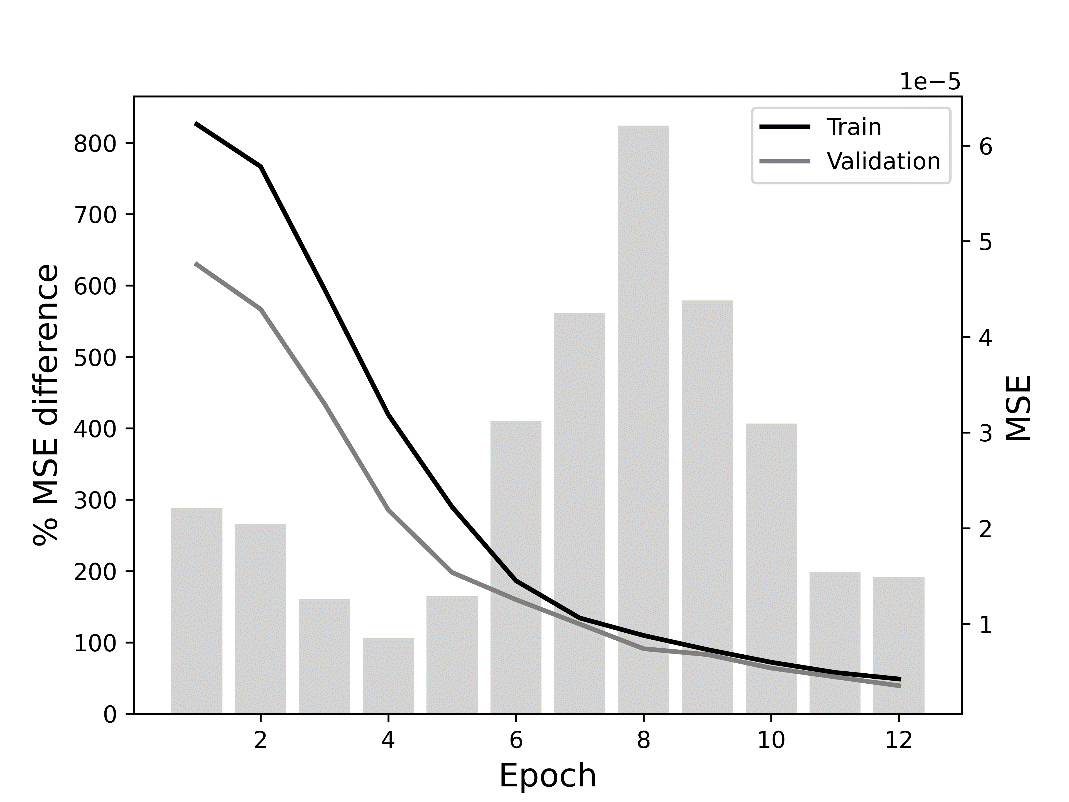


**Sup. Figure 1:** Metrics of the CAE fitting process. The bars represent the average percentage difference in MSE between artifact-free and artifact-laden images in dataset 1 (left y-axis). A high value is desirable. The line plots show the average MSE during fitting (right y-axis). A low value is desirable.


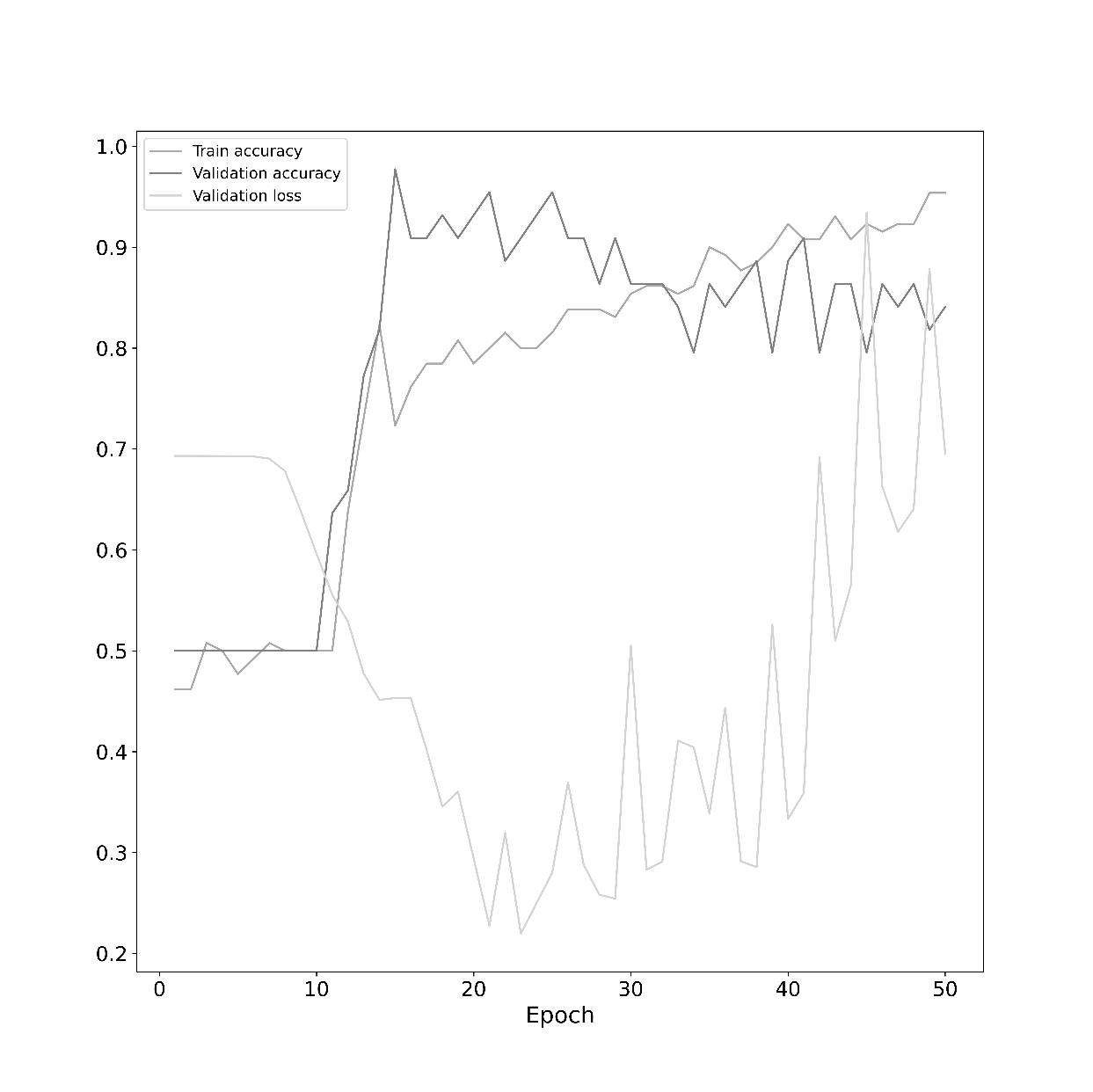


**Sup. Figure 2:** Metrics of the CNN fitting process. A number of 25 epochs was chosen.


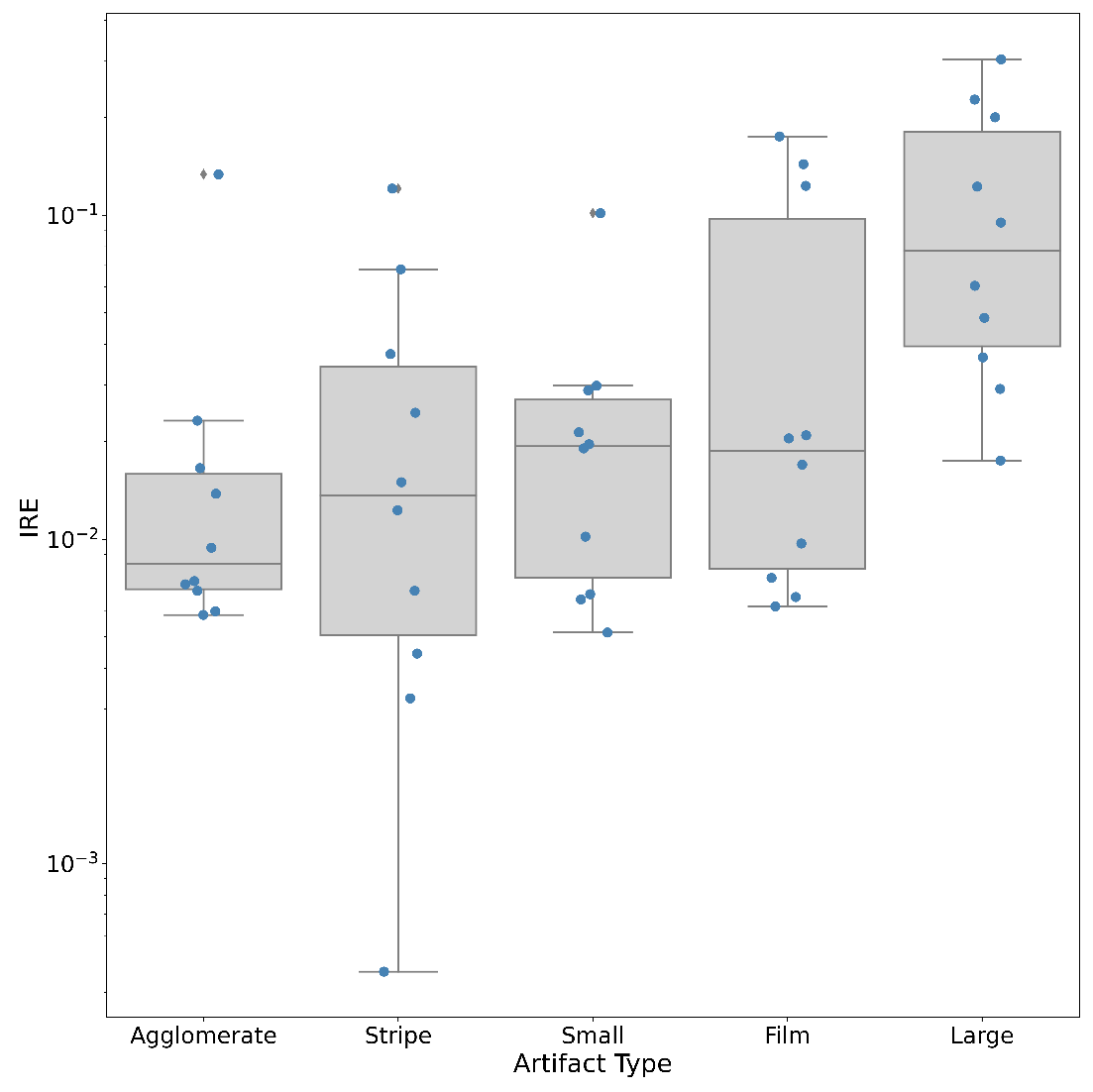


**Sup. Figure 3:** IRE values of different artifact types. Large artifacts resulted in the highest IRE values. Artifacts originating from surface films or those that are relatively small followed, with nearly identical median values. Stripes exhibited a wide variance in IRE values, with those producing only very weak signals and being largely or completely removed through preprocessing yielding the lowest values. Agglomerates resulted in the lowest IRE values overall. The images were collected from different experiments to obtain representative quantities for each class.


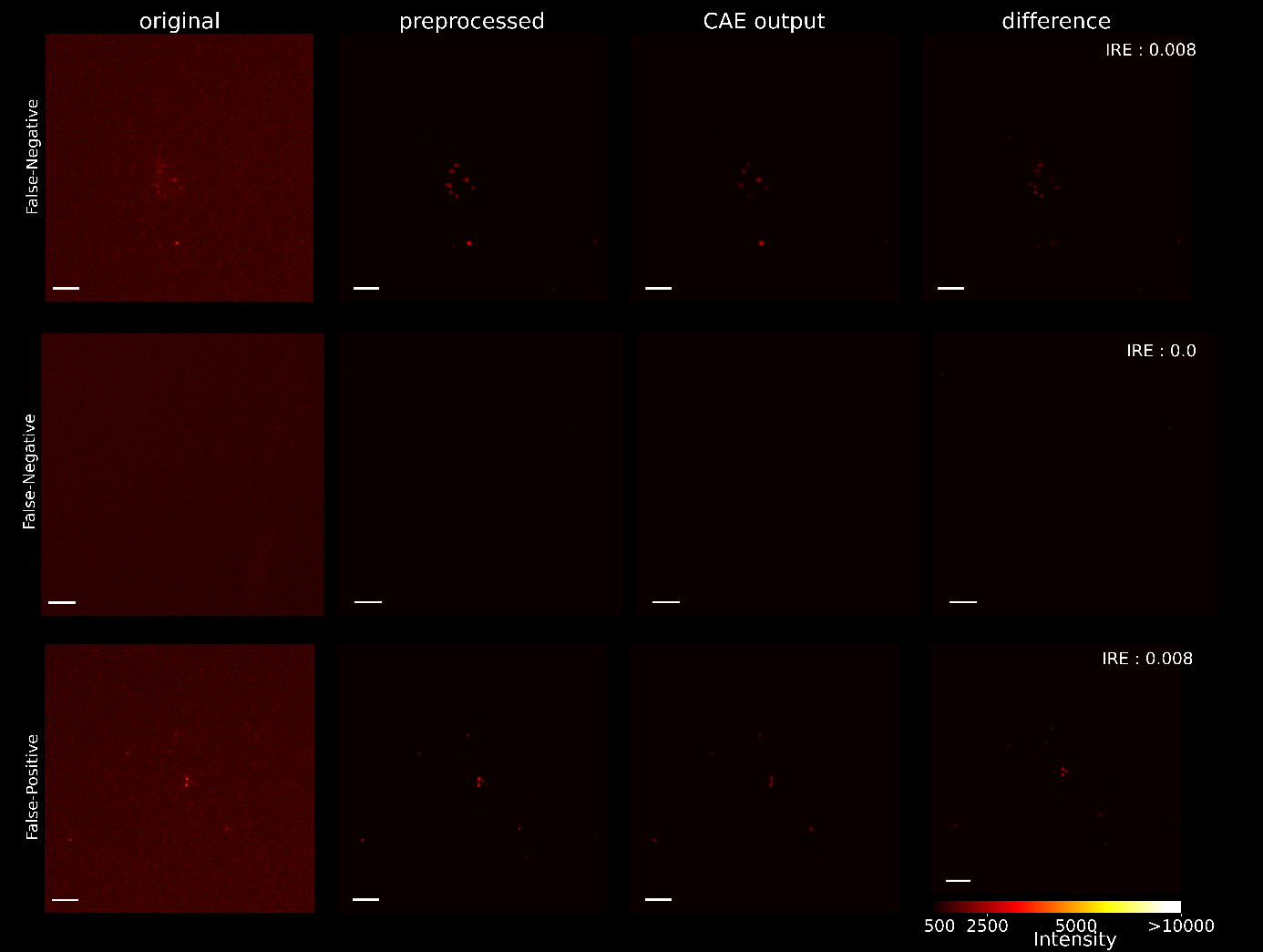


**Sup. Figure 4:** Some examples where the method fails. The first example shows an agglomerate consisting of very small particles. Due to the small size and the distance between the particles, no sufficient IRE is achieved. The second example shows a stripe with very low intensity. Due to the low intensity, it is removed during preprocessing. The third example shows two authentic signals that are very close to each other. It is important to note that the decision as to whether an image is considered artefact-laden depends on the dataset-specific IRE threshold.
